# Identification of Genetic Variants for Diabetic Retinopathy Risk Applying Exome Sequencing in Extreme Phenotypes

**DOI:** 10.1155/2024/2052766

**Published:** 2024-01-13

**Authors:** Juan C. Zenteno, Oscar F. Chacón-Camacho, Vianey Ordoñez-Labastida, Antonio Miranda-Duarte, Camila Del Castillo, Jessica Nava, Fatima Mendoza, Luis Montes-Almanza, Germán Mora-Roldán, Karlen Gazarian

**Affiliations:** ^1^Department of Genetics, Institute of Ophthalmology “Conde de Valenciana”, Mexico City, Mexico; ^2^Faculty of Medicine, Department of Biochemistry, National Autonomous University of Mexico (UNAM), Mexico City, Mexico; ^3^Rare Disease Diagnostic Unit, Faculty of Medicine, UNAM, Mexico City, Mexico; ^4^Laboratorio 5 Edificio A-4, Carrera de Médico Cirujano, Facultad de Estudios Superiores Iztacala, Universidad Nacional Autónoma de México, Mexico; ^5^Faculty of Medicine, Autonomous University of the State of Morelos (UAEM), Morelos, Mexico; ^6^Department of Genomic Medicine, Instituto Nacional de Rehabilitación “Luis Guillermo Ibarra Ibarra”, Mexico City, Mexico; ^7^Retina Department, Institute of Ophthalmology “Conde de Valenciana”, Mexico City, Mexico; ^8^Biomedical Research Institute, Department of Genomic Medicine, National Autonomous University of Mexico, Mexico City, Mexico

## Abstract

**Background:**

Diabetic retinopathy (DR) risk has been shown to vary depending on ethnic backgrounds, and thus, it is worthy that underrepresented populations are analyzed for the potential identification of DR-associated genetic variants. We conducted a case-control study for the identification of DR-risk variants in Mexican population.

**Methods:**

We ascertained 60 type 2 diabetes mellitus (T2DM) patients. Cases (*n* = 30) were patients with advanced proliferative DR (PDR) with less than 15 years after a T2DM diagnosis while controls (*n* = 30) were patients with no DR 15 years after the diagnosis of T2DM. Exome sequencing was performed in all patients, and the frequency of rare variants was compared. In addition, the frequency of variants occurring in a set of 169 DR-associated genes were compared.

**Results:**

Statistically significant differences were identified for rare missense and splice variants and for rare splice variants occurring more than once in either group. A strong statistical difference was observed when the number of rare missense variants with an aggregated prediction of pathogenicity and occurring more than once in either group was compared (*p* = 0.0035). Moreover, 8 variants identified more than once in either group, occurring in previously identified DR-associated genes were recognized. The p.Pro234Ser KIR2DS4 variant showed a strong protective effect (OR = 0.04 [0.001–0.36]; *p* = 0.04).

**Conclusions:**

Our study showed an enrichment of rare splice acceptor/donor variants in patients with PDR and identified a potential protective variant in *KIR2DS4*. Although statistical significance was not reached, our results support the replication of 8 previously identified DR-associated genes.

## 1. Introduction

Retinal effects of chronic diabetes mellitus (DM), both type 1 (T1DM) and type 2 (T2DM), involve severe vascular abnormalities, loss of the blood-retinal barrier, and neuronal damage leading to a clinical entity known as diabetic retinopathy (DR). DR is the most common cause of incident legal blindness in working age population, and it accounts for about 20% of new blindness incidents among patients who are 45–74 years old [1, 2]. About a third of diabetic people have signs of DR, and the estimated number of people with this condition will be 191 million by 2030 [3]. Importantly, DR is associated with enhanced risk of serious disabling systemic vascular complications, including stroke, coronary heart disease, and heart failure [4]. Clinically, DR is defined as the presence of typical retinal microvascular abnormalities in an individual with DM and is classified as nonproliferative diabetic retinopathy (NPDR) and proliferative diabetic retinopathy (PDR) according to the modified Airlie House Classification used in the Early Treatment Diabetic Retinopathy Study (ETDRS) [5]. NPDR includes the formation of microaneurysms and blot hemorrhages resulting from defects in retinal blood flow and vascular permeability, thickening of the basement membrane, and loss of pericytes [6]. PDR is characterized by pathologic retinal neovascularization, with vitreous hemorrhage, vitreous new blood vessels, and retinal traction detachment, which results in profound visual loss [7].

Currently established risk factors for the development of DR include diabetes duration, elevated HbA1c, glycemia, blood pressure, serum cholesterol, and low-density lipoprotein levels [8]. However, evidence for a strong genetic contribution has been obtained from twin and family studies, with heritability scores ranging from 25% to 52% for PDR in either T1DM or T2DM [9, 10]. The notion that genetic factors are central for DR development has been stressed by studies showing that levels of hemoglobin A1c (HbA1c) and disease duration account for only 11% of the retinopathy risk, and individuals with very well-controlled blood glucose levels may or may not develop DR [11]. Moreover, while DR usually has slow progression over decades after the initial diagnosis of diabetes, from mild to moderate to severe NPDR and finally most advanced PDR, some newly diagnosed or new-onset diabetic patients may develop NPDR or even PDR in a very short period [12, 13]. Available data supports the role of specific DNA variants in the predisposition to and natural history of DR [9, 10], and thus, identifying DR-risk variants has the potential to provide a more rational understanding into the molecular pathogenesis of the disease and to propose molecular targets for DR treatment or prevention.

In past years, the application of genome-wide association studies (GWAS) has allowed the recognition of several genetic variants that influence the risk of developing DR [14–17]. Unfortunately, the genotyping platform of traditional GWAS is inherently limited to common variants. Consequently, rare variants of large effect size and potential biologic relevance will be missed. Recently, exome sequencing (ES) has emerged as a cost-effective strategy for extending these studies to include rare coding variants, which could have more significant functional consequences. To date, a few studies have been performed using ES for the identification of DR-associated variants [18–20].

With a prevalence of 16.9%, or one in six adults, Mexico is one of the countries with the highest number of diabetic patients (International Diabetes Federation, https://idf.org/our-network/regions-and-members/north-america-and-caribbean/members/mexico/). In 2021, it was estimated that 14 million adults in Mexico were living with diabetes (http://www.diabetesatlas.org) and a population-based survey in a southern region of the country found that 38.9% of adults aged 50 or older with diabetes had DR and 21.0% had PDR [21]. As DR risk has been clearly shown to vary depending on ethnic backgrounds [22], it is desirable that underrepresented populations are analyzed for the potential identification of DR-risk variants. Using an extreme phenotype approach, we applied ES to a group of 30 DR subjects and compared their results with a group of 30 diabetic non-DR subjects to identify differentially mutated genes. Our results add to the genetic characterization of diabetic retinopathy and illustrate the usefulness of exome sequencing for the identification or rare coding variants associated with risk for this prevalent diabetic microvascular complication.

## 2. Methods

### 2.1. Study Population

Diabetes was diagnosed according to the American Diabetes Association criteria (2020). Duration of diabetes was calculated from the time of the first diabetes diagnosis. Patients were excluded if they fulfilled the criteria of type 1 diabetes mellitus, as previously reported [23]. The diagnosis of PDR and its staging was made based on ophthalmoscopy and fluorescein angiography by experienced ophthalmologists. DR was classified as proliferative according to the ETDRS criteria [5]. The presence of microaneurysms, hemorrhages, cotton wool spots, intraretinal microvascular abnormalities, hard exudates, venous beading, and new vessels was compatible with a PDR diagnosis. All patients had T2DM and were of Mexican mestizo ethnicity. Cases were defined as patients with advanced PDR in at least one eye and with less than 15 years after a T2DM diagnosis. Controls were patients with no DR in either eye with at least 15 years after the onset of T2DM. Written informed consent was obtained from all patients. Ethics approval for this study was obtained from the Faculty of Medicine Ethics Committee (117/2019).

### 2.2. Exome Sequencing

Genomic DNA was extracted from a 3-5 mL sample of venous blood. Genomic DNA isolation was performed with the QIAamp DNA Mini Kit (Qiagen), following the manufacturer's instructions. The obtained DNA was resuspended in 200 microliters of water, quantified with a NanoDrop equipment, and stored in the DNA Bank at -20°C for later use. Whole exome sequencing of samples was performed as an external service at the 3billion company (Seoul, South Korea). Briefly, exonic regions of all human genes (~22,000 genes) were captured with the xGen Exome Research Panel v2 kit (IDT, Coralville, Iowa, USA). The libraries were quantified by fluorimetry and were sequenced in a NovaSeq 6000 equipment (Illumina, San Diego, CA, USA) at a minimum depth of 50x. The analysis of the raw sequencing data, including alignment to the reference genome GRCh37/hg19 and the calling and annotation of variants, was performed with free access software and with the EVIDENCE program (3billion Inc.).

Once the sequencing files were received, we proceeded to identify and classify variants of clinical importance using the Franklin platform (https://franklin.genoox.com/clinical-db/home); this annotation database contains several human population databases as gnomAD (http://gnomad.broadinstitute.org/), the 1000 Genomes Project (http://browser.1000genomes.org), and dbSNP (http://www.ncbi.nlm.nih.gov/snp), as well as *in silico* prediction algorithms as SIFT (http://sift.jcvi.org), FATHMM (http://fathmm.biocompute.org.uk), and MutationAssessor (http://mutationassessor.org). Disease and phenotype databases as OMIM (http://www.omim.org), ClinVar (http://www.ncbi.nlm.nih.gov/clinvar), HGMD (http://www.hgmd.org), and HPO (https://hpo.jax.org/app/) were also employed for variant prioritization.

### 2.3. Exome Data Analyses

Four main analyses comparing allelic frequencies of variants between the PDR (cases) and non-DR (controls) group were performed:

1. Rare missense, stop gain/loss, frameshifting, and splice acceptor/donor site variants with a minor allele frequency of <1% in data from gnomAD and the 1000 Genomes Project
2. Rare missense, stop gain/loss, frameshifting, and splice acceptor/donor site variants with a minor allele frequency of <1% in data from gnomAD and the 1000 Genomes Project and observed more than once in either the PDR or non-DR group
3. Rare missense variants identified more than once in either the PDR or non-DR group and which had in silico aggregated prediction of pathogenicity according to the Franklin platform (https://franklin.genoox.com/clinical-db/home). Pathogenic aggregated prediction was defined as a score of ≥0.7 obtained from the analysis of ensemble methods such as REVEL and MetalR which use up to 13 different in silico tools to assign a prediction (for description, see https://help.genoox.com/en/articles/4341424-prediction-tools-and-score-range). In addition, the frequency of variants which were classified as pathogenic by 5 different predictors (SIFT, POLYPHEN, MUTATION ASSESSOR, MUTATIONTASTER, and FATHMM) and classified into “pathogenic (P)” or “likely pathogenic (LP)” in accordance with the American College of Medical Genetics and Genomics (ACMG) guidelines was also compared
4. Variants occurring in a set of 169 genes previously associated with DR [18–20, 24] (see supplementary data for gene list (available here)) identified more than once in either the PDR or the non-DR group and with significant different frequencies between both groups

### 2.4. Variant Quality Control

Only variants that passed the quality filters were included in the analyses. Variants with call rates less than 80% and variants with low mean depth of data (less than 15x) were excluded. Human reference genome build hg19 from the UCSC Genome Browser was employed.

### 2.5. Statistical Analyses

For rare variants, defined as <1% MAF in gnomAD and the 1000 Genomes Project databases, we examined variants that were found in more than one case in the PDR or non-DR group. Corrected chi-square statistics was applied to evaluate whether the frequency of selected rare variants was associated with DR phenotypes by comparing our DR group with diabetic controls without DR. The alpha level was 0.05, and the STATA ver. 15.0 statistical software package was used for calculations.

## 3. Results

**Figure 1:**
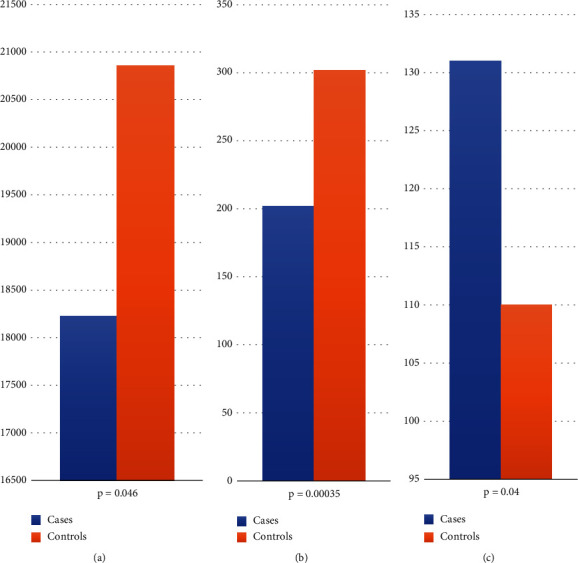
Variant frequencies showing statistically significant differences among groups. (a) Missense variants with population frequency less than 1%. (b) Missense variants with a population frequency less than 1%, which were found in at least two subjects, either in cases or controls, and which had in silico aggregate prediction of pathogenicity. (c) Splice acceptor/donor variants with a population frequency less than 1% and observed in at least two subjects, either in cases or controls.

We included 30 cases (16 females, 14 males) with a mean age at the beginning of the study of 56 years (range of 34 to 75 years), as well as 30 controls (20 females, 10 males) with a mean age of 66 years (range of 43 to 80 years).  Figure 1 shows the comparisons which exhibited statistical significant differences among groups.

### 3.1. Analysis #1

ES identified a total of 31,049 variants in cases and 35,079 in controls. The frequency of rare (<1% MAF) missense variants was 18,225 in cases and 20,589 in controls, which had a statistically significant difference (*p* = 0.046). Similarly, a statistical difference was observed in the frequency of rare splice acceptor/donor variants between cases (*n* = 273) and controls (*n* = 256) (*p* = 0.03). No significant differences were observed for frameshift or stop gain/loss variants (Table 1).

### 3.2. Analysis #2

A statistical difference was identified for the frequency of rare splice acceptor/donor variants occurring in at least two individuals in either group (*n* = 131 in cases and *n* = 110 in controls; *p* = 0.04). No differences were observed for rare missense, frameshift, or stop gain/loss variants (Table 2).

### 3.3. Analysis #3

A strong statistical difference was observed between groups when rare missense variants with pathogenic aggregated prediction and occurring in at least two individuals in either group were compared (202 in cases vs. 302 controls; *p* = 0.00035) (Table 3). A total of 19 rare variants with predicted pathogenicity in all five employed in silico tools (SIFT, Polyphen, Mut Assesor, MutationTaster, and FATHMM) and occurring more than once in either group were recognized; however, both the individual and global frequencies of such variant did not have statistical differences (41 variants in cases vs. 30 variants in controls; *p* = 0.097; Table 4). Finally, when the frequency of rare variants classified into “pathogenic (P)” or “likely pathogenic (LP)” in accordance with the ACMG guidelines, with predicted pathogenicity in all five employed in silico tools (SIFT, Polyphen, Mut Assesor, MutationTaster, and FATHMM), and occurring in at least two individuals in either group was compared, no differences were observed (6 variants in cases vs. 4 in controls; *p* = 0.48).

### 3.4. Analysis #4

The frequency of variants occurring in at least two individuals in either group, with significant differences between cases and controls, and occurring in a group of 169 genes previously associated with diabetic retinopathy [18–20, 24] was compared. Thus, from the 50 candidate genes identified by Song et al. [20], we identified 5 variants, located in 5 of such genes (*KMT2C*, *AHNAK2*, *DNAH10*, *KIR2DS4*, and *PAPSS2*). From the 43 DR-candidate genes identified by Ung et al. [19], 2 variants located in 2 different genes (*VPS13B* and *CFAP74*) were identified. From the 73 DR genes analyzed by Gu et al. [24], variants in only 1 gene (*HLA-DRB1*) was identified. Finally, no variants in the 3 candidate genes reported by Shtir et al. [18] were recognized in our analysis. Only the novel c.700C>T variant in *KIR2DS4*, predicting a p.Pro234Ser missense replacement, was shown to have statistically significant differences between groups as it occurred in 13 controls and in a single case and conferred a strong protective effect against PDR (odds ratio [95%confidence intervals] = 0.04 [0.001–0.36]; *p* = 0.04). While a trend to an increased PDR risk was observed for *VPS13B* c.5501C>T (p.Ser1834Leu) (OR: 7.25 [0.77–344.67]), no statistical significance was reached. Of the 8 variants identified in the set of 169 (~5%) DR-candidate genes, 4 were more common in our cases while the remaining 4 were more common in our controls (Table 5). Interestingly, one of the identified variants, c.381+2_381+3insAAAA in the *PAPSS2* gene, has been recently associated with DR in a Chinese cohort [20].

## 4. Discussion

With the advent of NGS, a more comprehensive characterization of exomic or genomic variants involved in complex traits development has been obtained, overcoming the inherent technical limitations of GWAS. ES offers an exceptional approach to identify the potential involvement of rare (coding) functional variants in human complex phenotypes. The identification of genetic variants conferring risk to DR is fundamental for a better understanding of the molecular events leading to this serious diabetic complication and for the early identification of individuals with a high visual loss risk. In this study, we applied ES to a group of 30 DR subjects and to a group of 30 diabetic non-DR subjects to identify differentially mutated genes and to compare the frequency of rare variants in genes previously associated with DR. In this cohort, statistically significant differences among groups were observed for total number of rare missense variants (18,225 in cases vs. 20,589 in controls; *p* = 0.046), for rare missense variants with pathogenic aggregated prediction (202 in cases vs. 302 controls; *p* = 0.00035), and for splice acceptor/donor variants (131 in cases vs. 110 in controls; *p* = 0.04), occurring in at least two individuals from either group.

To our knowledge, only three studies using exome sequencing for the identification of DR-associated variants have been previously published [18–20]. In 2016, Shtir et al. [18] analyzed a cohort of 43 diabetics who did not develop DR a decade or more after diagnosis (cases) and 64 diabetics with DR (controls) of Saudi origin; three genes (*NME3*, *LOC728699*, and *FASTK*) reached gene-based genome-wide significance and were considered as candidate DR-protective genes. In 2017, Ung et al. [19] analyzed 57 patients with PDR (cases) and 13 patients with no diabetic retinopathy despite at least 10 years of T2DM (controls), including individuals of African American descent and individuals of mixed ethnicities. After filtering for genes with null alleles in greater than two cases, 44 candidate genes were identified, including rare nonsynonymous variants in *FAM132A*, *SLC5A9*, *ZNF600*, and *TMEM217*. More recently, Song et al. [20] studied 15 subjects covering three extreme phenotypes of T2DM: the early-onset (DR) group (*n* = 6) included patients who developed DR within a median time of 1 year after the onset of T2DM, the non-DR group (DM; *n* = 5) included subjects who had no DR at least 10 years after the onset of T2DM, and the late-onset DR group (DM–DR; *n* = 4) included patients who had the first diagnosis of DR at least 10 years after the onset of T2DM. Through strict filtering (mutation rate difference ≥ 60% among comparison groups), a total of 54 genes were identified to exhibit significant differences. In the present study, we compared the frequency of rare variants occurring in at least two individuals from either group in 169 DR-associated genes recognized in such previous studies and identified rare variants in ~5% of those genes (8/169, including *AHNAK2*, *CFAP74*, *DNAH10*, *HLA-DRB1*, *KIR2DS4*, *KMT2C*, *PAPSS2*, and *VPS13B*). Although differences in frequencies did not reach statistical significance for 7 out of 8 of such variants, their observation more than once in either group could support their potential involvement in DR risk and warrants their additional investigation in larger DR cohorts. Interestingly, the particular *PAPSS2* c.381+2_381+3insAAAA variant, previously associated with DR [20], was observed to occur more frequently in our control group. *PAPSS2* encodes human PAPS synthase 2, a highly conserved enzyme involved in sulfur metabolism and in sulfation of proteins, glycans, and steroid hormones [25]. In humans, *PAPSS2* pathogenic variants originate several disorders including skeletal dysplasia, androgen excess, and polycystic ovary syndrome [26]. At this moment, no obvious role of this gene in DR is apparent and additional studies will be needed to replicate this association. In addition, a previously unrecognized variant at *KIR2DS4* was shown to occur predominantly in the control group, suggesting its role as a DR-protective variant (OR = 0.04 [0.001–0.36]; *p* = 0.04). *KIR2DS4* encodes a protein pertaining to the activating killer immunoglobulin-like receptor family which binds a variety of HLA ligands including HLA-C1 and HLA-C2 alleles [27]. To our knowledge, no association between *KIR2DS4* variants and risk for diabetic complications has been previously reported.

The present study included only Mexican individuals, a population mainly composed of mestizos, who are individuals with a genetic background consisting of Amerindian, European, and, to a lesser extent, African ancestries [28]. Ethnic groups with multiple ancestral origins, along with other non-European populations, have largely been underrepresented in genomic case-control study designs [29]. The mixture of ancestries present in admixed populations provides unique opportunities for the identification of novel gene-phenotype associations in complex traits as DR. On the other hand, we applied an “extreme phenotype” approach for variant discovery. The rationale of this approach is that extreme phenotypes will occur in individuals with an excess of rare variants, and thus, it is aimed at identifying rare genetic variants causing a large effect on disease risk [30]. Thus, in our work, we included patients with advanced PDR in at least one eye and with less than 15 years after a T2DM diagnosis and controls who were individuals with no PDR in either eye after at least 15 years of the onset of T2DM.

In our study, the frequency of rare splice acceptor/donor variants occurring in at least two subjects from either group showed statistical differences (131 in cases vs. 110 in controls; *p* = 0.04) and suggests the potential involvement of splicing defects in DR risk. The identification of dysregulated splicing profiles opens the opportunity for novel diagnostic and therapeutic tools in DR, as suggested for cancer [31]. However, as not all variants affecting splicing patterns will necessarily be deleterious [32], additional analyses of these results are warranted.

While our study included a small number of participants, the analysis strategy was mainly performed considering total number of variants which allowed the identification of significant differences. In fact, according to the number of variants in both study groups, we reached a high statistical power (above 80%). Finally, although our study has a clear limitation consisting in its small sample size, our results support the involvement of previously DR-associated genes and add evidence which could be analyzed in larger case-control studies. Hence, further validation of these results in additional DR populations and exploration of its biological implications in the disorder are warranted.

## Figures and Tables

**Table 1:** Total number of variants with population frequencies (gnomAD) less than 1% (rare variants).

Type of variant	Cases (*n* = 30)	Controls (*n* = 30)	Pearson's chi-squared test
Missense	18225	20859	0.046
Frameshift	775	862	0.74
Stop gain/loss	549	588	0.36
Splice acceptor/donor	273	256	0.03
Synonymous	11227	12514	0.19
Total	31049	35079	

**Table 2:** Total number of variants with population frequencies (gnomAD) less than 1% which occurred in at least two subjects, either in cases or controls.

Type of variants	Cases (*n* = 30)	Controls (*n* = 30)	Pearson's chi-squared test
Missense	8838	9600	0.38
Frameshift	494	537	0.93
Stop gain/loss	361	381	0.73
Splice acceptor/donor	131	110	0.04
Total	9824	10628	

**Table 3:** Total number of rare missense variants (<1% in gnomAD) which were observed in at least two subjects, either in cases or controls, and which had in silico aggregate prediction of pathogenicity.

Cases	Controls	Pearson's chi-squared test
202	302	0.00035

**Table 4:** List of 19 rare missense variants (<1% in gnomAD) which had prediction of pathogenicity in 5 predictors (SIFT, Polyphen, Mut Assesor, MutationTaster, and FATHMM) and were observed more than once, either in cases or controls.

	Variants	Cases (*N* = 30)	Controls (*N* = 30)	Pearson's chi-squared test	Fisher's exact test
1	ABCA5c.2612T>C	2	1	0.55	1.00
2	ABCA8c.3856C>T	0	2	0.15	0.49
3	ABCB5c.3100C>T	2	0	0.15	0.49
4	ABCB6c.739C>T	6	3	0.27	0.47
5	ACADVLc.68G>A	1	3	0.30	0.61
6	ALOX15Bc.932C>T	2	2	1.00	1.00
7	CACNA1Sc.1670G>A	3	1	0.30	0.61
8	CATSPER4c.247A>G	0	2	0.15	0.49
9	CHRNDc.117C>G	1	4	0.16	0.35
10	CNN2c.797G>A	2	2	1.00	1.00
11	COL27A1c.793G>A	0	2	0.15	0.49
12	DNASE1c.460C>G	7	2	0.70	0.14
13	DTHD1c.2621G>A	3	0	0.07	0.23
14	DUOX1c.2176G>A	2	0	0.15	0.49
15	EHD2c.838C>T	2	2	1.00	1.00
16	KCNJ14c.324C>G	2	1	0.55	1.00
17	NCF1c.269G>A	2	1	0.55	1.00
18	POTEJc.2077G>C	2	0	0.15	0.49
19	UNC93Ac.883G>A	2	2	1.00	1.00
		41	30	0.097	

**Table 5:** Variants identified from a set of 169 previously identified DR-associated genes [18–20, 24] and occurring more than once in either case or controls.

Gene	DNA variant	Protein variant prediction	Frequency in gnomAD (aggregated)	Case (*n*)	Control (*n*)	*p* ^†^	rs number
*KMT2C*	c.2537C>T	p.Ala846Val	0.142%	4	0	0.11	rs574432367
*AHNAK2*	c.7050C>T	p.Ala2350=	0.5037%	4	0	0.11	rs148170366
*DNAH10*	c.8281-10_8281-9delTT	—	0.4561%	4	0	0.11	rs34756279
*VPS13B*	c.5501C>T	p.Ser1834Leu	0.7958%	6	1	0.10	rs144257406
*KIR2DS4*	c.700C>T	p.Pro234Ser	N/A	1	13	0.0004	**—**
*CFAP74*	c.573G>A	p.Val191=	0.9187%	0	4	0.11	rs77196972
*HLA-DRB1*	c.33C>T	p.Cys11=	0.0125%	0	4	0.11	rs34396110
*PAPSS2* ^‡^	c.381+2_381+3insAAAA	—	N/A	0	4	0.11	rs367885911

^†^2-sided Fisher's exact. ^‡^Identified in the study by Song et al. [20].

## Data Availability

The VCF file data used to support the findings of this study are available from the corresponding author upon request.

## Supplementary Materials

Supplementary Materials Variants previously associated with DR occurring in a set of 169 genes.
