# Supplementary material for: Identification of Genetic Variants for Diabetic Retinopathy Risk Applying Exome Sequencing in Extreme Phenotypes
Source: Biomed Res Int. 2024 Jan 13;2024:2052766. doi: 10.1155/2024/2052766 (PMC10799704; doi:10.1155/2024/2052766)
Supplement: Supplementary Materials — Variants previously associated with DR occurring in a set of 169 genes. [file 2052766.f1.docx]

**Gu et al. 2020**

1. ACE NM_000789.4
2. AGER NM_001136.5
3. AGTR1 NM_000685.5
4. AHSG NM_001622.4
5. ANGPT1 NM_001146.5
6. ANGPT2 NM_001118887.2
7. ANGPTL4 NM_139314.3
8. AQP1 NM_198098.4
9. AQP4 NM_001650.7
10. B2M NM_004048.4
11. BDNF NM_001709.5
12. CCL2 NM_002982.4
13. CNTF NM_000614.4
14. CXCL10 NM_001565.4
15. CXCL8 NM_000584.4
16. EDN1 NM_001955.5
17. EDN3 NM_207034.3
18. EDNRA NM_001957.4
19. FBLN1 NM_006486.3
20. GDNF NM_000514.4
21. GFAP NM_002055.5
22. GSR NM_000637.5
23. HGF NM_000601.6
24. HIF1A NM_001530.4
25. HLA-A NM_002116.8
26. HLA-B NM_005514.8
27. HLA-DRB1 NM_002124.4
28. HSPB1 NM_001540.5
29. ICAM1 NM_000201.3
30. IDO1 NM_002164.6
31. IL10 NM_000572.3
32. IL17A NM_002190.3
33. IL2RA NM_000417.3
34. ITLN1 NM_017625.3
35. LCN1 NM_002297.4
36. LCN2 NM_005564.5
37. LPA NM_005577.4
38. MMP9 NM_004994.3
39. MTHFR NM_005957.5
40. NAGLU NM_000263.4
41. NAMPT NM_005746.3
42. NGF NM_002506.3
43. NPPA NM_006172.4
44. NTF3 NM_001102654.2
45. NUCB2 NM_005013.4
46. PDGFA NM_033023.5
47. PDGFB NM_002608.4
48. PGF NM_002632.5
49. PPARGC1A NM_013261.5
50. PRKCA NM_002737.3
51. PTGES NM_004878.5
52. PTGIS NM_000961.4
53. PTGS2 NM_000963.4
54. PTX3 NM_002852.4
55. RARRES2 NM_002889.4
56. RBP3 NM_002900.3
57. SEMA3A NM_006080.3
58. SEMA3E NM_012431.3
59. SERPINF1 NM_002615.7
60. SORD NM_003104.6
61. SORL1 NM_003105.6
62. SST NM_001048.4
63. TAC1 NM_003182.3
64. TGFB1 NM_000660.7
65. THBS1 NM_003246.4
66. TLR4 NM_138554.5
67. TNF NM_000594.4
68. TNFRSF1A NM_001065.4
69. TNFRSF1B NM_001066.3
70. UTS2 NM_006786.4
71. VCAM1 NM_001078.4
72. VEGFA NM_003376.6
73. VIP NM_003381.4

**Shtir et al., 2015**

1. FASTK NM_006712.5
2. NME3 NM_002513.2
3. PDE3A NM_000921.5

**Song et al. 2022**

1. ACLY NM_001096.3
2. AHNAK2 NM_138420.4
3. AIM2 NM_004833.3
4. ANKRD20A4
5. ATP8B3 NM_138813.4
6. C2orf81 NM_001316764.3
7. CRELD2 NM_024324.5
8. CUX2 NM_015267.4
9. DCAF17 NM_025000.4
10. DCHS2 NM_001358235.2
11. DNAH10 NM_001372106.1
12. DNAH17 NM_173628.4
13. DNAH3 NM_001347886.2
14. ECEL1 NM_004826.4
15. EFCAB2 NM_032328.4
16. ESX1 NM_153448.4
17. GP1BA NM_000173.7
18. GPRC5A NM_003979.4
19. HLA-H NR_001434.4
20. HLA-J NR_024240.1
21. IQGAP2 NM_006633.5
22. KIAA1841 NM_001129993.3
23. KIR2DS4 NM_001281971.2
24. KMT2C NM_170606.3
25. KRT10 NM_000421.5
26. MAMDC4 NM_206920.3
27. NLN NM_020726.5
28. NPHP3 NM_153240.5
29. PAPSS2 NM_001015880.2
30. PCNT NM_006031.6
31. PLCB3 NM_000932.5
32. POLR2J3 NM_001097615.2
33. POM121C NM_001099415.3
34. PRAMEF13 NM_001291380.1
35. PRAMEF22 NM_001100631.2
36. PRRC2A NM_004638.4
37. PTPRF NM_002840.5
38. RPL29 NM_000992.3
39. SEH1L NM_001013437.2
40. SETDB1 NM_001366418.1
41. SIGLEC1 NM_023068.4
42. SKOR2 NM_001278063.4
43. SLC18B1 NM_052831.3
44. SPATA31A3 NM_001083124.1
45. SPATA31C1 NM_001145124.1
46. TBC1D1 NM_015173.4
47. TGM1 NM_000359.3
48. VPS13D NM_015378.4
49. WDR61 NM_025234.3
50. ZNF322 NM_024639.5

**Ung et al. 2017 2017**

1. ABCA7 NM_019112.4
2. ABHD17A NM_001130111.2
3. AKR1C3 NM_003739.6
4. ANO2 NM_001364791.2
5. ATP12A NM_001676.7
6. BPIFB6 NM_174897.2
7. C15orf32 NR_161370.1
8. C1QTNF12 NM_001014980.3
9. CCDC105 NM_173482.3
10. CD96 NM_005816.5
11. CDKL1 NM_004196.7
12. CEP192 NM_032142.4
13. CFAP74 NM_001304360.2
14. COL6A5 NM_001278298.1
15. DNHD1 NM_144666.3
16. EFCAB3 NM_173503.4
17. FAM92A NM_145269.5
18. GPATCH1 NM_018025.3
19. HMCN1 NM_031935.3
20. HNRNPCL1 NM_001013631.3
21. KIF24 NM_194313.4
22. LRBA NM_001364905.1
23. LRP8 NM_004631.5
24. MPZL3 NM_198275.3
25. MSH2 NM_000251.3
26. NAT1 NM_000662.8
27. NLRP12 NM_144687.4
28. PHF21A NM_001352027.3
29. PKHD1L1 NM_177531.6
30. RGMA NM_020211.3
31. SIGLEC11 NM_052884.3
32. SLC5A9 NM_001011547.3
33. SLC6A13 NM_016615.5
34. SLURP1 NM_020427.3
35. TMEM217 NM_001286401.2
36. TTC22 NM_001114108.2
37. UPK3A NM_006953.4
38. VEGFB NM_003377.5
39. VPS13B NM_152564.5
40. ZDHHC11 NM_024786.2
41. ZDHHC11B NM_001351303.2
42. ZNF600 NM_001321866.3
43. ZNF77 NM_021217.3
